# Transcriptome Analysis of Duck Liver and Identification of Differentially Expressed Transcripts in Response to Duck Hepatitis A Virus Genotype C Infection

**DOI:** 10.1371/journal.pone.0071051

**Published:** 2013-07-29

**Authors:** Cheng Tang, Daoliang Lan, Huanrong Zhang, Jing Ma, Hua Yue

**Affiliations:** 1 College of Life Science and Technology, Southwest University for Nationalities, Chengdu, China; 2 College of Tibetan Plateau Research, Southwest University for Nationalities, Chengdu, China; Washington State University, United States of America

## Abstract

**Background:**

Duck is an economically important poultry and animal model for human viral hepatitis B. However, the molecular mechanisms underlying host–virus interaction remain unclear because of limited information on the duck genome. This study aims to characterize the duck normal liver transcriptome and to identify the differentially expressed transcripts at 24 h after duck hepatitis A virus genotype C (DHAV-C) infection using Illumina–Solexa sequencing.

**Results:**

After removal of low-quality sequences and assembly, a total of 52,757 unigenes was obtained from the normal liver group. Further blast analysis showed that 18,918 unigenes successfully matched the known genes in the database. GO analysis revealed that 25,116 unigenes took part in 61 categories of biological processes, cellular components, and molecular functions. Among the 25 clusters of orthologous group categories (COG), the cluster for “General function prediction only” represented the largest group, followed by “Transcription” and “Replication, recombination, and repair.” KEGG analysis showed that 17,628 unigenes were involved in 301 pathways. Through comparison of normal and infected transcriptome data, we identified 20 significantly differentially expressed unigenes, which were further confirmed by real-time polymerase chain reaction. Of the 20 unigenes, nine matched the known genes in the database, including three up-regulated genes (virus replicase polyprotein, LRRC3B, and PCK1) and six down-regulated genes (CRP, AICL-like 2, L1CAM, CYB26A1, CHAC1, and ADAM32). The remaining 11 novel unigenes that did not match any known genes in the database may provide a basis for the discovery of new transcripts associated with infection.

**Conclusion:**

This study provided a gene expression pattern for normal duck liver and for the previously unrecognized changes in gene transcription that are altered during DHAV-C infection. Our data revealed useful information for future studies on the duck genome and provided new insights into the molecular mechanism of host–DHAV-C interaction.

## Introduction

Duck is an economically important cultured poultry and animal model for human viral hepatitis B [1]–[3]. Duck viral hepatitis (DVH) is a highly contagious disease that occurs frequently in domestic ducklings with high mortality, causing great economic losses in the duck industry [4]. Traditionally, DVH is caused by three serotypes of RNA viruses, namely, DHV types I, II, and III. DHV Type I, the most common and internationally widespread type, is a member of Picornaviridae; the other two types (II and III) are classified as members of the Astroviridae [5], [6]. To better distinguish different DHVs among the members of Picornaviridae, DHV Type I was renamed duck hepatitis A virus (DHAV) [7]. Furthermore, DHAV was categorized into three genotypes, A (DHAV-A), B (DHAV-B), and C (DHAV-C), based on the genetic structures of DHAV [4]. DHAV-C is a recently found DHAV genotype that has caused serious damage to the local duck industry in China and South Korea because of its high mortality [8], [9]. Nevertheless, the molecular mechanisms involved in the pathogenesis of DHAV-C and the host-directed antiviral responses remain poorly understood because of limited information on the duck genome.

Transcriptome is a sum of all gene transcription products of a specific tissue or cell in a functional state, which is a link with the genetic information of genome and proteomic biology function. Transcriptome studies are the basis and starting point in the study of gene function and regulatory networks, and it is now being widely used in many fields including the host-pathogens interaction [10]–[12]. Recently developed high-throughput (HT) sequencing technologies, such as Illumina–Solexa sequencing technology, has provided a powerful tool for transcriptome analysis and bring great advantages over conventional methods [13] . This method enables analysis of the complexity of whole transcriptome and allows identification of millions of expressed genes especially when the genomic information is unknown. Comparing to the conventional methods such as microarrays, RNA-Seq has great advantages including less bias, a greater dynamic range, a lower frequency of false positive and higher reproducibility [14], [15]. It is being increasingly recognized that transcriptome sequencing is an efficient mean of characterizing the molecular basis of host–virus relationship [10], [11], [16], [17]. It also facilitates functional genomic studies, including profiling of global gene expression, assembly of full-length genes, and novel gene discovery, particularly when genome information is limited [18], [19]. To date, only one study has reported on the transcriptome sequencing of feather bulbs in ducks [20]. However, data on the gene expression profile of duck liver and its response to viral infection at the whole transcriptome level are lacking. In this study, transcriptome sequencing was applied to determine the gene expression pattern of duck liver tissue. A comparative analysis of transcriptome data between the normal control group and the DHAV-C infected group was performed. The results of this study may serve as a basis for future studies on the genetic architecture of the liver transcriptome and may facilitate the discovery of candidate genes that can respond and resist DHAV-C infection in ducks.

## Materials and Methods

### Experiment animals and virus infection

DHAV-C challenge experiment and sample collection were performed as previously described [8], [9]. In brief, six three-day-old specific pathogen-free ducks were divided into two groups. One group was infected with 0.2 mL DHAV-C strain swun 3504 (4.73×10^4^ copies) through hypodermic injection. The other group (mock group) was treated with the same dose of isotonic sodium chloride. Similar to previous studies, the clinical symptoms were observed at 24h post infection and the clinical course of infection of these indviduals in infected group was basically same. Liver tissue was collected from three ducks per group at 24 h post infection and was preserved in Sample Protector (Takara, China) at −80°C for RNA extraction. The animal experiment was approved by the Animal Disease Control Center of Si Chuan province, China (The approved permit number is SYXK2011-043). All ducks were handled in compliance with the local animal welfare regulations and maintained according to standard protocols.

### RNA extraction, cDNA library construction, and Illumina-Solexa sequencing

Total RNA of three ducks per group was extracted using TRIZOL reagent (Takara, China) according to the manufacturer's instructions. The RNA pool was prepared by mixing together equal quantities of three RNA samples per group. cDNA library preparation and Illumina–Solexa sequencing were performed as previously described [21]. In brief, Poly (A) mRNA was isolated from the total RNA with oligo(dT) magnetic beads and then fragmented into short pieces. Cleaved short RNA fragments were used for first-strand cDNA synthesis using reverse transcriptase and random hexamer–primer. Second-strand cDNA fragments were then synthesized using DNA polymerase I, dNTPs, and RNase H. After purification and paired-end (PE) repair, the cDNA fragments were ligated to sequencing adapters and amplified by polymerase chain reaction (PCR) to obtain the final PE library. The library was sequenced using an Illumina HiSeqTM 2000, and all the obtained data were submitted to the National Center for Biotechnology Information (NCBI) database Short Read Archive (accession number is SRA066082).

### Transcriptome Data Analysis

The raw reads were first cleaned by removing adaptor sequences, empty reads, and filtering reads with low quality (phred quality <5). The Trinity program (Table S1) (http://trinityrnaseq.sourceforge.net/) was used for the de novo assembly of the remaining reads. The reads were first combined to form longer fragments named contigs, then the reads were mapped back to contigs, and with paired-end reads it was able to detect contigs from the same transcript as well as the distances between these contigs. Finally, get sequences by connecting these contigs and cannot be extended on either end. Such sequences were defined as unigenes. ESTScan program (Table S1) was used to analyze the open reading frame (ORF) of the unigenes. All the unigenes were predicted and annotated using local BLAST programs against the NCBI nr, nt, SwissProt, STRING and Cluster of Orthologous Groups (COG) (Table S1) databases (10^−5^ E-value cutoff). Based on the results of the blast annotation, Blast2GO tool (Table S1) was applied to analyze the functional classification of the unigenes based on Gene Ontology (GO) (Table S1) terms, and WEGO tool (Table S1) was used to classify GO function. The pathway of the involved unigenes was annotated by performing local BLAST program (blastp)-mediated comparisons against the Kyoto Encyclopedia of Genes and Genomes (KEGG) (Table S1) database. Differentially expressed sequences between two libraries were identified by the programs RSEM (Table S1) (http://deweylab.biostat.wisc.edu/rsem/) [22] and edger (Table S1) (http://www.bioconductor.org/packages/elease/bioc/tml/edgeR.html) [23]. Briefly, the RSEM was used firstly to calculate the number of mapping reads to every assembled unigenes and estimate unigenes expression levels. Then EdgeR was used to trim the unigenes counts obtained by RSEM and analyses differential expression. The TMM(trimmed mean of M-values) method was selected to computing normalization factors [24], the negative binomial distribution was selected for calculate the p value, and Benjamini and Hochberg method was used for adjust multiple testing. Unigenes with p-values less than 0.05 and FDR less than 0.001 were identied as signicantly dierentially expressed.

### Verification by real-time PCR (qRT-PCR)

The differentially expressed genes were detected by real-time PCR to confirm the sequencing data. Information on the individual primer sequences of the 20 target genes and the internal reference gene (β-actin) is listed in Table 1. For real-time PCR, a SYBR®Premix Ex Taq™ II (Tli RNaseH Plus) Kit (Takara, Japan) and an ABI7500 FAST Real-time PCR System (ABI) were used according to the manufacturers' instructions. Reaction conditions were as follows: 95°C for 1 min, followed by 40 cycles of 95°C for 10 s and 60°C for 40 s. A final solubility curve analysis was completed. The relative expression of each gene was calculated based on a previously described 2^−ΔΔCT^ method [25].

**Table 1:** Primer sequences of the differentially expressed genes for real-time PCR analysis.

Gene name	Primer sequence (5′-3′)	Size
Replicase polyprotein	F-CACCGACCCGAACCTCTT	160bp
	R-CCTGTGAAACATACCCATCCA	
C-reactive protein(CRP)	F-AAGGGGATGAAATTGGGTGT	208bp
	R-TGGAGGAGCTTGAGCTACGA	
Activation-induced C-type lectin 2(AICL-like 2)	F-ATGGCAGTCAAGGAAGTATCAG	104bp
	R-GCAAGAAGACACCAGGAGTAGA	
Leucine rich repeat containing 3B(LRRC3B)	F-GCATGGCGTAATCGGTAGT	160bp
	R-CAGCAGGTGTTGAGGAGCA	
Neural cell adhesion molecule L1-like protein(L1CAM)	F-CGAGACCGATTGGAACGA	174bp
	R-GAAGGAGCAGCATCTGGAGT	
Cytochrome P450, family 26, subfamily A, polypeptide 1(Cyp26a1)	F-CTCTGGGACCTGTATTGCG	166bp
	R-GAGTCTTGTAGATGAAGCCGTAT	
Cation transport regulator-like protein 1(CHAC1)	F-TTTCCTTATGTCCCTTTCACTG	193bp
	R-AGACTTCATGCGCTACTTCTGT	
Phosphoenolpyruvate carboxykinase, cytosolic ,GTP ( PCK1)	F-ATAGCCTAACCAACCTAAGACCT	190bp
	R-GCTGACCTTCCCTACGAAAT	
Disintegrin and metalloproteinase domain-containing protein 32(ADAM32)	F-ACATCCCAAACCTGAGACCA	162bp
	R-ATAAAGAAAGCACTGAGGAGGAG	
Beta-actin (β-actin)	F-CATCCACGAAACTACCTTCAACT	164bp
	R-TGATTTTCATCGTGCTGGGT	
Unkown unigene (comp15974_c0)	F-ATCTTTGCTGTAACTCTTTGCC	115bp
	R-TCTACCCAGATGCTTACTTTGTT	
Unkown unigene (comp15791_c0)	F-GACAACACGCACATCACCA	143bp
	R-ACCAACAGCAGTCTTACATCG	
Unkown unigene (comp13083_c0)	F-TTCCCAGTGTCTCACAGAAATC	146bp
	R-GAGTAAAGTGATAGGCTGGAAGAT	
Unkown unigene (comp14937_c0)	F-TTGTTTCCGTAAGCCAAATAAT	124bp
	R-CTGAGGGTTTCTCCTTTCTGTT	
Unkown unigene (comp23_c0)	F-ATCTGGGCTGCCTCATCA	162bp
	R-ATCTCAAAATGTTTTCCCACTG	
Unkown unigene (comp10495_c0)	F-GCTTTGCTTGTTTCTTGCC	181bp
	R-AGATGATATTCCCAGGTTTGG	
Unkown unigene (comp8017_c0)	F-TTCTGCTGAGTTGGCACATT	156bp
	R-AAGCCCCAGATAAGGAAGC	
Unkown unigene (comp21253_c0)	F-ACAGTTGCCCACAGTTTCG	136bp
	R-CTCCCGTGTCAGCCAGTC	
Unkown unigene (comp15940_c0)	F-AGCTTTTCATCCCTACACCTATT	174bp
	R-AACACTCAAGTTTCCCATCCA	
Unkown unigene (comp17402_c0)	F-CTTGCCACTGCTTGTTCTTC	164bp
	R-AGGAGGCTGGACTACACGAC	
Unkown unigene (comp10077_c0)	F-ACATAAGCAGGCAGTTCAGACA	189bp
	R-CCAACCCCACCTCCAGTG	

## Results

### RNA-seq and assembly of transcriptome data from normal liver

Sequencing data from normal group was submitted to the NCBI database (accession number is SRA066082). After Illumina-Solexa deep sequencing, a total of 83,096,430 raw reads were obtained in the cDNA library of normal liver. The removal of low-quality reads (i.e., reads containing only adaptors and empty reads) resulted in 61,480,114 clean reads with total residues of 105,026,747 bp. After de novo sequence assembly, a total of 52,757 unigenes with an average length of 1,316.82 bp were generated. Analysis of nucleotide content showed that the overall guanine-cytosine (GC) content of the transcriptome was 45.5%. Among the total unigenes, further blast and ORF analyses showed that only 18,918 matched the known genes in the database and 12,141 were predicted ORF. A total of 2,213 unigenes have completed ORF in the remaining non-annotation unigenes.

### Function analysis of transcriptome Data from normal liver

**Figure 1:**
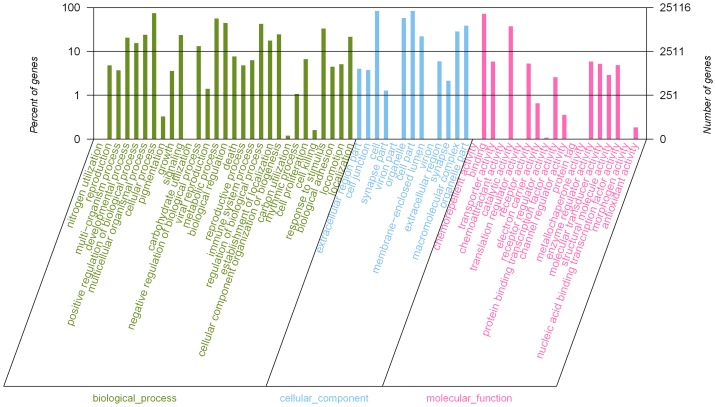
GO function classification of normal liver transcriptome.

**Figure 2:**
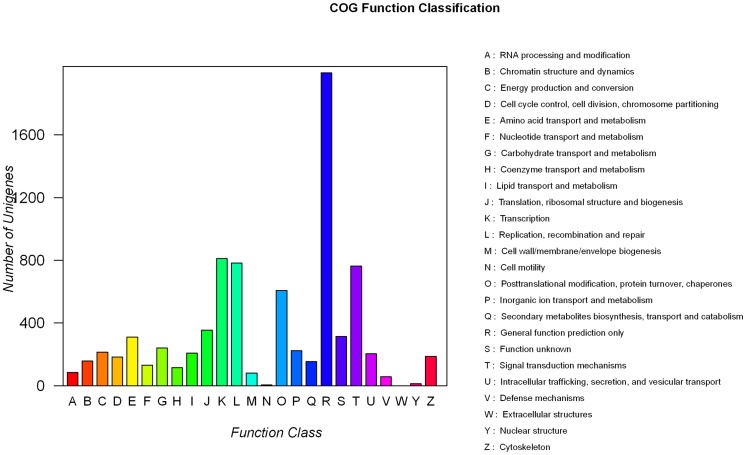
COG function classification of normal liver transcriptome.

The putative functions of unigenes in normal liver libraries was analyzed using GO and COG. Analysis of GO categories showed that 25,116 unigenes were mapped to 61 categories of biological processes, cellular components, and molecular functions (Figure 1). In the categories of biological processes, most of the corresponding genes were involved in cellular process, pigmentation, and growth. In the categories of cellular components, most of the corresponding genes were involved in cell junction, cell, and synapsis. In the categories of molecular functions, most of the corresponding genes were involved in chemorepellent, binding, and transporter activities. Among the 25 COG categories, the cluster for “General function prediction only” represented the largest group, followed by “Transcription” and “Replication, recombination, and repair” (Figure 2).

KEGG analysis showed that 17,628 unigenes were involved in 301 pathways. Partial innate immune-associated pathways are listed in Table 2. The Ubiquitin mediated proteolysis pathway accounted for the largest proportion in innate immune-associated pathways, which contained 332 unigenes. Three innate immune receptor pathways, Toll-like receptor (TLR), RIG-I-like receptor (RIG-I), and nucleotide oligomerization domain (NOD)-like receptor (NLR) signaling pathways, can also be found. In addition, many unigenes were involved in other innate immune-associated pathways, including T and B cell receptor signaling pathway, proteasome pathway, apoptotic pathway, and Jak-STAT signaling pathway.

**Table 2:** Partial innate immune-associated pathways in normal liver transcriptome.

PathWay	Pathway_definition	Number of unigenes
path:ko04120	Ubiquitin mediated proteolysis	332
path:ko04660	T cell receptor signaling pathway	210
path:ko04210	Apoptosis	209
path:ko04620	Toll-like receptor signaling pathway	184
path:ko04630	Jak-STAT signaling pathway	184
path:ko04350	TGF-beta signaling pathway	179
path:ko04666	Fc gamma R-mediated phagocytosis	174
path:ko04662	B cell receptor signaling pathway	174
path:ko04622	RIG-I-like receptor signaling pathway	157
path:ko04650	Natural killer cell mediated cytotoxicity	150
path:ko04621	NOD-like receptor signaling pathway	131
path:ko04115	p53 signaling pathway	115
path:ko04612	Antigen processing and presentation	66
path:ko03050	Proteasome	38

### Identification of differentially expressed genes

Sequencing data from infected group was submitted to the NCBI database (accession number is SRA066082). In the infected group library, a total of 70,845,824 raw reads were obtained after Illumina deep sequencing. The removal of low-quality reads resulted in 52,291,086 clean reads with total residues of 103,233,928 bp. After de novo sequence assembly, a total of 52,145 unigenes were generated. Comparison of normal and infected transcriptome data revealed that 20 unigenes were significantly differentially expressed between the two libraries (Table 3). Among these 20 differentially expressed unigenes, only nine unigenes can be annotated and the others did not match any known genes in the database. However, four unigenes were predicted to have completed ORF in the non-annotation unigenes. Among the nine annotated unigenes, one (replicase polyprotein gene) was from the virus, and the rest were from the host. Compared with the control library, five unigenes were significantly up-regulated in the infected library, including three known and two unknown unigenes (Table 3). Fifteen unigenes were significantly down-regulated, including six known and nine unknown unigenes (Table 3). KEGG analysis showed that only the cytochrome P450, family 26, subfamily A, polypeptide 1 (CYP26A1) and phosphoenolpyruvate carboxykinase gene 1 (PCK1) genes were involved in related pathways. The former was involved in retinol metabolism pathway, and the latter was involved in 10 pathways, including glycolysis/gluconeogenesis, adipocytokine signaling pathway, pyruvate metabolism, microbial metabolism in diverse environments, biosynthesis of secondary metabolites, metabolic pathways, insulin signaling pathway, proximal tubule bicarbonate reclamation, citrate cycle, and peroxisome proliferator-activated receptor signaling pathway.

**Table 3:** Differentially expressed unigenes during DHAV-C infection.

Gene	P Value	FDR	Up (+)/Down(−)	qRT-PCR fold change (Infection/normal )
Virus Replicase polyprotein	7.53E-15	3.83E-10	+	/^a^
C-reactive protein(CRP)	2.93E-14	7.46E-10	−	/^b^
Activation-induced C-type lectin 2(AICL-like 2)	6.44E-14	1.09E-09	−	0.00002
Leucine rich repeat containing 3B(LRRC3B)	1.69E-11	1.45E-07	+	34.3
Neural cell adhesion molecule L1-like protein(L1CAM)	1.71E-11	1.45E-07	−	0.027
cytochrome P450, family 26, subfamily A, polypeptide 1(Cyp26a1)	5.60E-09	2.59E-05	−	0.016
Cation transport regulator-like protein1 (CHAC1)	2.05E-08	8.04E-05	−	0.043
Phosphoenolpyruvate carboxykinase, cytosolic,GTP (PCK1)	2.80E-08	9.48E-05	+	24.25
Disintegrin and metalloproteinase domain-containing protein 32(ADAM32)	7.70E-08	0.000230297	−	0.049
Unkown unigene (comp15974_c0)	7.78E-13	9.89E-09	−	0.0042
Unkown unigene (comp15791_c0)	9.60E-11	6.97E-07	−	0.033
Unkown unigene (comp13083_c0)	4.69E-10	2.98E-06	−	0.0063
Unkown unigene (comp14937_c0)	5.48E-10	3.09E-06	−	0.00402
Unkown unigene (comp23_c0)	1.78E-09	9.06E-06	−	/^b^
Unkown unigene (comp10495_c0)	9.04E-09	3.83E-05	−	0.00054
Unkown unigene (comp8017_c0)	2.56E-08	9.31E-05	−	0.29
Unkown unigene (comp21253_c0)	4.09E-08	0.000130137	+	282.09
Unkown unigene (comp15940_c0)	1.47E-07	0.000414944	−	0.0031
Unkown unigene (comp17402_c0)	3.20E-07	0.000856951	+	7.78
Unkown unigene (comp10077_c0)	3.41E-07	0.000866835	−	/^b^

a. This unigene was only detected in DHAV-C infected group but not detected in normal group. b. These unigenes were only detected in normal group but not detected in DHAV-C infected group.

### Verification of differential gene expression by real-time PCR

Real-time PCR was performed to measure the expression of the 20 differentially expressed unigenes and confirm the differential gene expression from the transcriptome data. The replicase polyprotein gene was not detected in normal group. The C-reactive protein (CRP) gene and one Unkown unigene (comp10077_c0) were not detected in DHAV-C infected group (Table 3). The expression patterns of the remaining genes were correlated with the transcriptome data (Table 3), indicating reliability of the data.

## Discussion

At present, the molecular mechanisms underlying host–pathogen interaction remain unclear because of limited information on the duck genome. Several reports revealed that transcriptome sequencing is not only an efficient method for profiling genes whose expressions are altered during infection but also for obtaining new genomic information. DVH is a serious threat to domestic ducklings. New types of viral hepatitis have emerged in recent years because of intensive aquaculture and trade globalization. Recently, DHAV-C infection, a new genotype of DVH with high mortality, has spread in Southeast Asia, which caused great economic losses in the local duck industry [8], [9]. However, the molecular mechanisms underlying host antiviral strategies and DVH pathogenesis remain unclear because of limited information on the genome and gene expression patterns of duck liver. We used transcriptome sequencing to explore and compare the gene expression patterns of normal and infected duck livers. This study aims to describe the genetic architecture of the normal liver transcriptome and further facilitate investigations on the duck genome and molecular events during DHAV-C interaction.

### Genetic architecture of the normal duck liver transcriptome

The high-throughput sequence data obtained by Illumina-Solexa deep sequencing were used to understand the genetic architecture of the duck liver transcriptome. In this study, we pooled RNA from three normal healthy individuals to generate a sample. Deep sequencing was subsequently performed. After removal of low-quality sequences and assembly, we obtained 52,757 unigenes with an average length of 1,316.82 bp. Analysis of nucleotide content within all unigenes showed that the overall GC content of the transcriptome was 45.5%, which is close to the reported transcriptome data (47.02%) and genome-wide exons (47.00%) of chicken, but much higher than that of chicken genome-wide introns (40.00%). Given the limited data on the duck genome, we further blasted these unigenes using NCBI nr, nt, and SwissProt databases. The results showed that 18,918 unigenes matched the known genes in the database, whereas the remaining unigenes could not be annotated. However, 12,141 of the remaining non-annotated unigenes were predicted to have ORF, of which 2,213 have completed ORF. Although these unigenes did not match the known genes, some (if not all) may be new genes or ncRNA that can provide useful basis for novel genomic information on ducks. In addition, we further analyzed the 18,918 annotated unigenes by blasting the chicken genome. The result showed that only 14,883 annotated unigenes (78.7%) matched genes present in the chicken genome, suggesting that a large genetic difference exists between ducks and chickens.

To date, only one study has reported on the transcriptome data of duck feather bulbs [20]. Studies focusing on other duck tissues, including liver, are lacking. In this study, a genome-wide description of the genetic architecture of duck liver was provided using high-throughput transcriptome sequencing. Compared with the transcriptome data of duck feather bulbs, the liver transcriptome library has a larger data size, with more raw reads (83,096,430/5,000,000), clean tags (61,480,114/4,887,399), and matched unigenes (18,918/1,0467), which may attributed to the specificity of gene expression in different tissues. These data can provide useful information for further investigations on the duck genome.

### Analysis of differentially expressed genes

Through comparison of the transcriptome data of the two libraries, we identified 20 significantly differentially expressed unigenes after DHAV-C infection. Among these 20 differentially expressed unigenes, nine matched the known genes in the database, including replicase polyprotein, CRP, activation-induced C-type lectin 2 (AICL-like 2), leucine rich repeat containing 3B (LRRC3B), neural cell adhesion molecule L1 (L1CAM), Cyp26a1, cation transport regulator-like protein 1 (CHAC1), PCK1, and disintegrin and metalloproteinase domain-containing protein 32 (ADAM32), all of which are host genes, except for the replicase polyprotein gene, which is a viral gene. Replicase polyprotein, a conserved protein in many RNA virus, contains the activities necessary for the transcription of the viral RNA genome [26], [27]. In the present study, the expression of the replicase polyprotein gene of DHAV-C was up-regulated in the infected group, whereas it was not be detected in the control group. This result showed that the host liver was infected with DHAV-C successfully, during which DHAV-C started to reproduce. However, common innate immune related genes, such as Toll-like receptors, Nod-like receptors, and interferon (IFN) genes, were not screened in 20 differentially expressed genes. In addition, the novel non-annotated unigenes accounted for a large proportion of the differentially expressed unigenes (11/20, 55%), which may provide a clue for the discovery of new transcripts associated with immune and infection in duck. This observation may serve as a basis for further investigation of new transcripts associated with duck infection.

An important characteristic of many viral infections is the induction of host cell apoptosis, which is an important means of host resistance to the virus [28]. However, apoptosis inhibition also exists in many viral infections, which is conducive to viral replication through the extension of the survival time of infected cells [29]–[31]. A previous apoptotic morphological study revealed that DHAV-C infection could cause apoptosis and necrosis in different tissues, especially in the liver [9]. However, the molecular mechanism of apoptosis induced by DHAV-C infection remains unclear. In the present study, two novel genes (CHAC1 and CYP26A1) associated with apoptosis were screened. CHAC1, a highly conserved protein between mammals and bacteria, is a new member of the unfolded protein response (UPR) pathway, which is a stress-signaling pathway in the endoplasmic reticulum [32]. It acts as a pro-apoptotic component of the unfolded protein response pathway by mediating the pro-apoptotic effects of the ATF4-ATF3-DDIT3/CHOP cascade [33]. In this study, CHAC1 expression was significantly down-regulated in the infected group. The repression of CHAC1 expression may inhibit host apoptosis to facilitate DHAV-C replication in viral infection.

In addition to CHAC1, another apoptosis-related gene, CYP26A1, was also detected. As an isoform of the CYP26 family, CYP26A1 is a major enzyme that controls retinoic acid (RA) homeostasis by catalyzing RA into bio-inactive metabolites [34]. It is moderately induced by RA in numerous tissues but is highly responsive in liver [35]. Recent studies have shown that the cells expressing CYP26A1 gain significant resistance to apoptosis because of the state of reduced bioavailability caused by the metabolic inactivation of RA [36]. This finding supports the idea that CYP26A1 expression levels may play a role in determining cellular commitment to apoptosis. In this study, the expression of CYP26A1 in the liver was significantly down-regulated in the infected group. DHAV-C infection may induce an anti-apoptotic mechanism to facilitate viral propagation by suppressing CYP26A1 expression to the extension of RA biological activity. These results indicated that apoptosis resistance and apoptosis both existed during DHAV-C infection, proving that host–virus interaction is a dynamic process.

Innate immune antiviral response first identifies pathogens through the pathogen-associated molecular pattern receptors and then releases the related cytokine to induce an antiviral immune reaction. In this study, the common innate immune recognition receptors, such as TLRs, NLRs, and RIG-I-like receptors, were not screened. Only one member of the C-type lectin receptor gene family, AICL-like 2, was detected. AICL-like 2, is a member of the natural killer (NK) receptor subfamilies of C-type lectin. It was first found as an activation-induced receptor encoded by the human NK gene complex, predominantly in NK and hematopoietic cells [37]. Other subfamily members of C-type lectin receptors, such as DC-SIGN, L-SIGN and Dectin-1, recognize the pathogens as the innate immune receptor [38]. In addition, DC-SIGN and L-SIGN have been proven to be major receptors of virus entry, which promote efficient viral replication in filoviruses and HIV infection [39], [40]. However, the exact function of AICL-like 2 and its pathogen recognition ability remains unknown. Recent studies have shown that Kaposi's sarcoma-associated herpesvirus can escape the attack of NK cell-mediated cytotoxicity by down-regulating the expression of C-type lectin receptors in NK cells, including AICL [41]. The down-regulation of AICL may also suppress tumor necrosis factor production by infected monocytes [42]. In this study, the expression level of AICL-like 2 was significantly down-regulated in the infected group, indicating that NK cell evasion may also exist in DHAV-C infection and may contribute to the ability of the virus to provide a reservoir for viral replication and transmission within the lesion.

IFN is a cytokine with a wide spectrum of antiviral and immunoregulatory functions. IFN plays an important role in the host resistance to infection. However, direct IFN and IFN signaling genes were not screened. Only one novel IFN related to the LRRC3B gene was detected in this study. LRRC3Bis a novel gene cloned from lymphocytic cells in relapsed leukemia [43]. Previous studies showed that LRRC3B is regulated by DNA methylation [44] and plays an important role in DNA repair [45]. Recent studies have proven that it could be a putative tumor suppressor gene in many types of cancer in human [46]. Interestingly, the transfection and expression of LRRC3B in gastric cancer cell line showed that immune response-related genes and IFN signaling genes can be activated effectively [47], suggesting that LRRC3B may be involved in immune-related processes that trigger the innate immune response through activation of the IFN signaling pathway. However, the gene involved in virus infection has not been reported. In the present study, the expression of LRRC3B was significantly up-regulated in the infected group compared with the control group. The high expression of LRRC3B indicates that the host resisted the virus through the induction of immune response. However, the direct immune-related genes were not screened in this study. These findings may be attributed to the infection time and IFN signaling genes not induced in this infection stage.

Aside from the above four known genes, other four genes (CRP, L1CAM, ADAM32, and PCK1) with different features were also screened. CRP is an acute phase protein produced by hepatocytes predominantly in response to inflammation and infection [48]. The level of CRP is correlated with the infection progress and could be considered as a disease progression prognostic factor in many types of hepatitis virus infection in human, including hepatitis A, B, and C [49]–[52]. Interestingly, the present study showed that the expression of CRP was significantly down-regulated in the infected group. This finding agrees with several study results previously reported in human hepatitis C infection. CRP levels were proven to be significantly lower in HCV+ patients than in HCV- patients [53]. Therefore, DHAV-C infection could promote a disturbance in CRP production.

L1CAM (also known as CD171), a member of the immunoglobulin super family, is a type I transmembrane glycoprotein that mediates calcium-independent cell–cell adhesion [54]. L1CAM has long been characterized as a cell recognition molecule with an important role in the development of the nervous system [54]. Aside from the nervous system, recent studies have shown that L1CAM regulates the transendothelial migration and trafficking of dendritic cells (DCs). Under inflammatory conditions, the expression of L1CAM could enhance the transmigration of DCs [54]. In this study, the expression level of L1CAM was significantly down-regulated in the infected group. This phenomenon may be related to the resistance of the virus to host immunity by inhibition of transendothelial migration and trafficking of the DCs to escape the host antigen presentation.

ADAM32 is a member of the ADAM family. ADAMs are a family of transmembrane and secreted proteins that play important roles.in many biological processes, including sperm–egg interactions, cell migration, axon guidance, and muscle and nervous system development [55]. A recent study has shown that ADAM family members, as key responders to TGF-β1, could induce lesion-like idiopathic pulmonary fibrosis in alveolar epithelial cells under several stimulating conditions, including Epstein Barr virus infection [56]. ADAM32 is predominantly expressed in the testis and may play a role in sperm development or possibly fertilization [57]. However, its involvement in other biological processes is still unclear. In this study, the expression of ADAM32 was significantly declined compared with the control group, suggesting the deregulation of ADAM32 in DHAV-C infection. An important characteristic in DHV is liver injury, and the down-regulated expression of ADAM32 may be associated with the host reduce infection lesion.

PCK1 (also known as PECK) is a rate-limiting enzyme that catalyzes the first committed step in gluconeogenesis. Hepatocytes play an important role in maintaining glucose homeostasis by regulating the balance between the hepatic gluconeogenic and glycolytic pathways [58]. PCK1 catalyzes a rate-controlling step of gluconeogenesis, which has become an important marker for hepatic gluconeogenesis. Recent studies have proven that the expression level of PCK1 is closely related to blood glucose control and diabetes development. Human Hepatitis C virus infection also causes liver diseases and extrahepatic manifestations, including metabolic disorders [59]. HCV promotes hepatic gluconeogenesis by significantly up-regulating the transcription of PCK1, leading to insulin resistance and type 2 diabetes in predisposed individuals [60]. In this study, the expression of PCK1 was significantly up-regulated in the virus infected group, which agrees with the results of previous studies in hepatitis C infection. The result indicates that hepatic gluconeogenesis, which leads to glucose abnormalities, also existed in DHAV-C early infection.

In summary, we explored the gene expression patterns in normal duck liver and the changes in gene transcription that are altered during DHAV-C early infection using de novo transcriptome sequencing. A total of 52,757 non-redundant unigenes were obtained, which contribute greatly to future studies on the duck genome. In addition, 20 differentially expressed unigenes (9 known genes and 11 novel unigenes) were screened during DHAV-C infection, which may serve as a basis for the discovery of new transcripts associated with infection and may provide new insights into the molecular mechanism of host–DHAV-C interaction in DHAV-C infection.

## Supporting Information

Table S1 Glossary of transcriptome data analysis tools. A short introduction refers to the transcriptome data analysis tools used in the present study.
(DOC)
