# Supplementary material for: Transcriptome Analysis of Duck Liver and Identification of Differentially Expressed Transcripts in Response to Duck Hepatitis A Virus Genotype C Infection
Source: PLoS One. 2013 Jul 29;8(7):e71051. doi: 10.1371/journal.pone.0071051 (PMC3726580; doi:10.1371/journal.pone.0071051)
Supplement: Table S1 — Glossary of transcriptome data analysis tools. A short introduction refers to the transcriptome data analysis tools used in the present study. (DOC) [file pone.0071051.s001.doc]

**Table S1. Glossary of transcriptome data analysis tools**

| Tools | Meaning |
| --- | --- |
| ESTScan | A software package for analyze the open reading frame (ORF) of the assembled sequences of RNA-Seq. |
| Trinity | A software package for assembly of the clean reads of RNA-Seq into contigs. |
| Blast2GO | A comprehensive bioinformatics tool for functional annotation of sequences and data mining on the resulting annotations, primarily based on the gene ontology (GO) term. |
| WEGO | A useful tool for classify GO annotation results. It has become one of the daily tools for downstream gene annotation analysis, especially when performing comparative genomics tasks. |
| [RSEM](https://github.com/bli25wisc/RSEM) | A software package for estimating gene and isoform expression levels from RNA-Seq data. |
| EdgeR | A software package for differential expression analyses of RNA-Seq. |
| GO | Short for Gene Ontology, which is an international standardized gene functional classification system which offers a dynamic-updated controlled vocabulary and a strictly defined concept to comprehensively describe properties of genes and their products in any organism. GO has three ontologies: molecular function, cellular component and biologicalprocess. The basic unit of GO is GO-term. Every GO-term belongs to a type of ontology. |
| COG | Short for the database of Clusters of Orthologous Groups of proteins, which is an attempt on phylogenetic classification of the proteins encoded in complete genomes. Each COG includes proteins that are inferred to be orthologs (direct evolutionary counterparts). |
| KEGG | Major public pathway-related database. It is used in our pipeline to perform pathway enrichment analysis of DEGs. This analysis identifies significantly enriched metabolic pathways. |
